# Different Proportions of Huangqi (*Radix Astragali Mongolici*) and Honghua (*Flos Carthami*) Injection on **α**-Glucosidase and **α**-Amylase Activities

**DOI:** 10.1155/2015/785193

**Published:** 2015-03-22

**Authors:** Hui Liao, Linda Banbury

**Affiliations:** ^1^Department of Pharmacy, Shanxi Provincial People's Hospital, Taiyuan 030012, China; ^2^Department of Pathogenic Biochemistry, Institute of Natural Medicine, Toyama University, Toyama 930-0194, Japan

## Abstract

*Objective.* To study the effect of different proportions of Huangqi (*Radix Astragali Mongolici*) and Honghua (*Flos Carthami*) injection on *α*-glucosidase and *α*-amylase activity simultaneously.* Methods.* The injections were prepared according to the standards of the China Food and Drug Administration. The assay for potential *α*-glucosidase inhibitors was based on the hydrolysis of 4-methylumbelliferyl-*α*-D-glucopyranoside (4-MUG). The *α*-amylase EnzChek assay kit was used to determine potential *α*-amylase inhibitors. Acarbose was the positive control.* Results.* The half maximal (50%) inhibitory concentration (IC_50_) of acarbose against *α*-glucosidase and *α*-amylase was (1.8 ± 0.4) *μ*g/mL and (227 ± 32) *μ*g/mL, respectively. Honghua showed significant inhibition of *α*-glucosidase activity compared with Huangqi (*P* < 0.01). Honghua inhibited *α*-amylase activity, but Huangqi did not. IC_50_s for *α*-glucosidase inhibition by mixtures at 10 : 1, 5 : 1, and 2 : 1 were significantly lower than those at the 20 : 1 mixture (*P* < 0.01). *α*-Amylase inhibition by the 2 : 1 mixture was significantly higher than that by the 20 : 1, 10 : 1, and 5 : 1 mixtures at 500 *μ*g/mL and 1000 *μ*g/mL (*P* < 0.01), with 5 : 1 significantly higher than 20 : 1 and 10 : 1 at 1000 *μ*g/mL (*P* < 0.01).* Conclusion.* Honghua significantly inhibited *α*-glucosidase activity compared with Huangqi (*P* < 0.01). For simultaneous inhibition of *α*-glucosidase and *α*-amylase activities, the mixtures at 2 : 1 and 5 : 1 exhibited significant effects compared with those at 20 : 1 (*P* < 0.01).

## 1. Introduction 

Carbohydrates in foods are digested into oligosaccharides under the action of salivary and pancreatic *α*-amylases and then digested into monosaccharides by *α*-glucosidase at the brush borders of small intestinal mucosal cells. The monosaccharides are absorbed by small intestinal epithelial cells in the blood circulation. To seek new opportunities in the treatment of diabetes mellitus, a number of studies have been carried out in China and abroad on inhibitors of the two enzymes. Currently, microorganisms and plants are the main sources of inhibitors of the two enzymes [1, 2]; for example, acarbose, which is commonly used clinically, is derived from microbial metabolites.

In this study we sought *α*-glucosidase and *α*-amylase inhibitors from the traditional Chinese medicines commonly used for treating diabetes mellitus [2], based on the modern Chinese medicine theory that the underlying pathogenesis of diabetes mellitus and its complications is blood stasis due to* qi* deficiency [3] and on clinical reports that Huangqi (*Radix Astragali Mongolici*) injection was usually combined with Honghua (*Flos Carthami*) injection to treat diabetes mellitus and its complications [4]. In clinical usage, there are different reports about the mixture proportions of Huangqi and Honghua injection, ranging from 4 : 1 to 10 : 1 [4–6]. In this research, Huangqi and Honghua injection were extracted according to the national standards established by the China Food and Drug Administration (CFDA). The contents of astragaloside IV in Huangqi injection and total flavonoids in Honghua injection were the quantitative requirements in the CFDA standards, and we determined these two active ingredients separately. The aim of this research was to study the effects of Huangqi and Honghua injection on *α*-glucosidase and *α*-amylase activity and to determine their optimum mixture proportions to inhibit these two enzymes simultaneously.

## 2. Methods

### 2.1. Preparation of Samples

Huangqi (*Radix Astragali Mongolici*) and Honghua (*Flos Carthami*) were purchased from Shanxi Double-Crane Pharmaceutical Co., Ltd., and were identified by the Department of Pharmacy of Shanxi Provincial People's Hospital. The Huangqi injection was prepared as follows [7]. Huangqi (2000 g) was added into water and decocted three times, for 1.5 h each time. The decocted solutions were combined and filtered. The filtrate was concentrated to a solution containing 1.6 g raw herb per mL. Ethanol was added twice: first to achieve an alcohol content of 75% and then alcohol content of 85% in the second time; the filtrate was kept at 4°C–8°C for 12 h each time. The solution was collected and filtered. Ethanol was removed from the filtrate, which was then concentrated to a solution containing 10 g raw herb per mL, and then was diluted with water for injection to 0.87 g raw herb per mL. The solution was refrigerated, set aside for 12 h, and then filtered. The filtrate was concentrated to a solution containing 5.6 g raw herb per mL and was adjusted to pH 7.5 with 20% sodium hydroxide solution. Activated carbon (0.125%) was added to the filtrate and it was boiled for 5 min and the mixture was stirred well, filtered, ultra-filtered, and then was adjusted to pH 7.5 again with 20% sodium hydroxide solution. Water for injection was added to a total volume of 1000 mL, thereby formulating an extract solution containing 2 g of raw herb per mL.

The Honghua injection was prepared as follows [8]. Honghua (500 g) was added into water and decocted three times: for 1 h, then for 50 min, and lastly for 30 min. The decocted solutions were combined and filtered. The filtrate was concentrated to a relative density of 1.20, and ethanol was added to achieve an alcohol content of 70%. After being refrigerated at 4°C for 48 h, the solution was filtered. Ethanol was removed from the filtrate, which was then concentrated to a relative density of 1.10. Additional ethanol was added to achieve an alcohol content of 80%, and the solution was filtered after it was refrigerated and set aside for another 48 h. Ethanol was removed from the filtrate, which was then concentrated to a relative density of 1.16. Thereafter, a 10-fold volume of water was added, and the solution was refrigerated, set aside for 20 h, and then filtered. The filtrate was concentrated to a relative density of 1.02 and was adjusted to pH 7.5 with 20% sodium hydroxide solution. It was heated at 115°C for 15 min, refrigerated, and set aside for 120 h. An appropriate amount of activated carbon was added, and the mixture was stirred well, filtered, ultra-filtered, and then was adjusted to pH 7.5 again with 20% sodium hydroxide solution. Water for injection was added to a total volume of 1000 mL, thereby formulating an extract solution containing 0.5 g of raw herb per mL.

A 10 mL aliquot of the above Huangqi injection solution was accurately pipetted and dried* in vacuo* to a constant weight. The resultant extract was weighed as 208.60 mg and the yield was calculated as 10.43 mg of extract per gram of raw herb. A 5 mL aliquot of the above Honghua injection solution was accurately pipetted and dried* in vacuo* to a constant weight. The resultant extract was weighed as 260.90 mg and the yield was calculated as 104.36 mg of extract per gram of raw herb. The extract solution and the dried extracts were both stored at 0°C–4°C until use.

### 2.2. Reagents and Instruments

*α*-Amylase EnzChek Assay Kit and fluorescein-conjugated corn starch (DQ starch) were from PerkinElmer (Boston, Mass, USA). Porcine pancreatic *α*-amylase (type VI-B), yeast *α*-glucosidase (EC3.2.1.20), 4-methylumbelliferyl-*α*-D-glucopyranoside (4-MUG), kaempferol, and astragaloside IV were from Sigma-Aldrich China (Shanghai, China). Acarbose (Glucobay, 50 mg/tablet) was obtained from Bayer Health Care Company Ltd. (Beijing, China). Methanol and aluminium muriate, 50 mM sodium acetate buffer (pH 5.5), 100 mM sodium glycinate buffer (pH 10.6), and 50 mM 3-morpholinopropanesulfonic acid buffer (MOPS, pH 6.9) were all prepared in-house from reagents purchased from Wako Chemicals USA Inc. (Richmond, VA, USA). The Wallac 1420 Victor2 plate reader, the 96-well polystyrene microplates, black 96-well microplates and the DELFIA 1296-003 plate shaker were purchased from PerkinElmer (Boston, MA, USA).

### 2.3. Determination of Astragaloside IV in Huangqi Injection [7]

Preparation of the control solution: an appropriate amount of astragaloside IV as a control was weighed accurately, and then methanol was added to prepare a solution containing 1 mg of astragaloside IV per mL as a control solution. Preparation of the test solution: 25 mL of Huangqi injection was measured accurately, dried to a constant weight, and then supplemented with methanol to 5 mL. 8, 12, and 16 *μ*L of the control solution and 10 *μ*L of the test solution were accurately pipetted and injected into a liquid chromatograph, respectively. The concentration of the test sample was obtained by calculating the common logarithm of the concentration based on the common logarithms of the peak areas and concentrations of the control solutions by the external standard method and converting it into the content of the test sample.

### 2.4. Determination of Total Flavonoids in Honghua Injection [8]

Preparation of reference solution: a reference solution of kaempferol was prepared in methanol at 0.1 mg/mL. Preparation of standard curve: 1.0, 2.0, 3.0, 4.0, 5.0, and 6.0 mL of the reference solution were added into 25 mL volumetric flasks and made up to volume with methanol. After shaking, 2.0 mL of each of the above solutions was added into a 10 mL test tube with a stopper. Then 1.0 mL of 0.1 M aluminium trichloride solution and 2.0 mL of methanol were added into the test tube, shaken well, and heated in a water bath at 40°C for 20 min. The test tube was removed from the water bath and cooled down to room temperature. With a corresponding reagent as the blank, the absorbance at wavelength of 422 nm was measured by UV-Vis spectrophotometry as described in Appendix VA of the Chinese Pharmacopoeia 2010, Volume I. A standard curve was plotted using the absorbance values as the *y* axis and the concentrations as the *x* axis.

Determination of total flavonoids: 0.5 mL of the Honghua injection extract solution was added into a 50 mL volumetric flask, made up to volume with methanol, and shaken well (test solution). 2.0 mL of the test solution was added into a 10 mL test tube with a stopper and its absorbance was measured according to the method as described in the preparation of standard curve. The blank solution was prepared by the same steps as above except that it contained 2.0 mL of the test solution and 3.0 mL of methanol. The total flavonoid content, as kaempferol equivalents in the test solution, was calculated after comparison with the kaempferol standard curve.

### 2.5. Stability of Different Mixtures of Huangqi Injection and Honghua Injection

According to the normal descriptions of the different mixtures' proportions based on the raw herbs and the range from 4 : 1 to 10 : 1 in clinical reports [4–6], we tested the stability of different mixture proportions of Huangqi injection and Honghua injection as follows: 20 : 1, 10 : 1, 5 : 1, and 2 : 1. Proportion 20 : 1 was 10 mL Huangqi injection mixed with 2 mL Honghua injection, mixed well, and tested for the pH value, particulate determination, and UV spectrum at 0, 1, 2, 3, and 5 h. Other proportions were tested as for 20 : 1, and the stability trial results were listed in Table 1.

### 2.6. Determination of Effect of Huangqi Injection, Honghua Injection, and Mixtures of These Two on Yeast *α*-Glucosidase Activity

A fluorimetric assay was used for the screening of potential yeast *α*-glucosidase inhibitors. The assay was based on 4-methylumbelliferyl-*α*-D-glucopyranoside (4-MUG), the substrate, being hydrolysed by *α*-glucosidase to yield the fluorescent product, 4-methylumbelliferone (4-MU) [9].

*α*-Glucosidase group: a 1 mM stock substrate solution of 4-MUG was diluted with 50 mM sodium acetate buffer to give a final assay concentration of 84 *μ*M. The substrate (45 *μ*L) was added to 96-well plates containing 50 *μ*L *α*-glucosidase and 5 *μ*L sodium acetate buffer. The contents of the microplate were mixed on an orbital shaker for 30 s and incubated for 20 min at 37°C. The reaction was stopped by the addition of 100 mM sodium glycinate (100 *μ*L). The plate was shaken for further 30 s, and the fluorescence measured at *λ*_ex_ 355 nm and *λ*_em_ 460 nm.

For the test samples and positive control, instead of 5 *μ*L buffer, 5 *μ*L samples or acarbose was added to wells and subsequent assay steps were carried out as above. The test concentrations of Huangqi were as follows: 375, 750, 1500, and 2000 *μ*g/mL and Honghua: 15.6, 31.3, 62.5, and 125 *μ*g/mL. The concentrations of mixtures with Huangqi and Honghua proportions 20 : 1, 10 : 1, 5 : 1, and 2 : 1 were all as follows: 31.3, 62.5, 125, and 250 *μ*g/mL. Acarbose was the positive control, and its concentration was 0.3, 0.6, 1.3, and 2.5 *μ*g/mL. Sodium acetate buffer (50 *μ*L/well) was used as a negative control. Each concentration was assayed 6 times

(1) α-glucosidase  inhibition  % =100×Aα-glucosidase−Asamples  or  acarboseAα-glucosidase−Anegative.

The 50% inhibitory concentrations (IC_50_ values) of the samples and acarbose on *α*-glucosidase activity were calculated based on the active concentrations and the inhibitory effects.

### 2.7. Determination of Effect of Huangqi Injection, Honghua Injection, and Mixtures of These Two on Yeast *α*-Amylase Activity

The measurement method as described by Omichi and Ikenaka [10] and the product information brochure for Molecular Probes EnzChek Amylase Assay Kit (E-11954) were used with a minor modification. A mammalian *α*-amylase enzyme was used instead of the bacterial enzyme provided in the kit. The reaction is based on the principle that the substrate fluorescein-conjugated corn starch (DQ starch) is not fluorescent but hydrolysis of DQ starch by *α*-amylase can produce degraded fragments with high fluorescence. The presence of *α*-amylase inhibitors can reduce the hydrolysis of DQ starch, thereby decreasing the production of the fluorescent fragments. The effects of the samples on *α*-amylase can be reflected by the changes in fluorescence before and after addition of the test samples.

The “*α*-amylase group” (*α*-amylase with no inhibitors present) was obtained by adding 95 *μ*L of 125 U/mL *α*-amylase solution, 10 *μ*L of 50 mM MOPS buffer, and finally 95 *μ*L of 1 mg/mL DQ starch, vortexing the above mixture for 30 s and incubating it at 37°C for 30 min. The fluorescence values were measured at *λ*_ex_ 505 nm and *λ*_em_ 512 nm.

Each test group was prepared by the same procedure as above except for replacing the buffer with 10 *μ*L of the test samples. The test concentrations of Huangqi and different proportions were all at 500 and 1000 *μ*g/mL, and Honghua was at 125, 250, 500, and 1000 *μ*g/mL. For the acarbose positive control, buffer was replaced with 10 *μ*L acarbose, and the test concentrations were 62.6, 125, 250, and 500 *μ*g/mL. The negative control was prepared by the same procedure as above except for replacing *α*-amylase with 95 *μ*L of buffer. The measurement was repeated six times

(2) α-amylase  inhibiyion  % =100×Aα-amylase−Asamples  or  acarbose    Aα-amylase−Anegative.

### 2.8. Statistics

Statistical analysis was done using SPSS software (version 12.0, SPSS Inc., Chicago, IL, USA). All data were expressed as mean ± standard deviation. Student's* t*-test was used for intergroup comparison. *P* < 0.05 was considered statistically significant.

## 3. Results

### 3.1. Content of Astragaloside IV in Huangqi Injection and Total Flavonoids in Honghua Injection

According to the standards of CFDA, Huangqi injection solution contains 2 g of raw herb per mL and the content of astragaloside IV is no less than 0.08 mg per mL. In our research, astragaloside IV was 0.127 mg per mL injection, equivalent to 6.1 *μ*g per mg extract and 63.5 *μ*g astragaloside IV equivalents per g dried raw herb.

According to the new standards established in 2013, Honghua injection solution contains 0.5 g of raw herb per mL and the content of total flavonoids in 1 mL injection is 0.20–0.70 mg. Our result showed that the total flavonoids were 0.57 mg per mL, equivalent to 10.9 *μ*g kaempferol per mg extract and 1.14 mg total flavonoid equivalents per g dried raw herb.

### 3.2. Stability of Different Mixtures of Huangqi Injection and Honghua Injection

The results showed that there were no significant differences in pH value, particulate determination, and UV spectrum among the different proportions and different time points (data not shown). Corresponding injection solution, extract, and active ingredients were also listed in Table 1.

### 3.3. Inhibitory Effect of Huangqi Injection, Honghua Injection, and Mixtures on Yeast *α*-Glucosidase Activity

In Table 2, the IC_50_ value of acarbose was (1.8 ± 0.4) *μ*g/mL, Huangqi was (1686 ± 810) *μ*g/mL, and Honghua was (32.8 ± 5.7) *μ*g/mL. There was a significant difference among them by Paired* t*-test (*P* < 0.01). IC_50_ values of the different mixtures, 20 : 1, 10 : 1, 5 : 1, and 2 : 1, were all significantly lower than Huangqi alone (*P* < 0.01) and significantly higher than Honghua alone (*P* < 0.01). IC_50_ values of 10 : 1, 5 : 1, and 2 : 1 did not show any significant difference among them by Paired* t*-test (*P* > 0.05), but they all were significantly lower than those of the 20 : 1 mixture (*P* < 0.01).

### 3.4. Inhibitory Effect of Huangqi Injection, Honghua Injection, and Mixtures on *α*-Amylase Activity

Acarbose showed an inhibitory effect on *α*-amylase activity from 62.5 *μ*g/mL to 500 *μ*g/mL, with an IC_50_ value of (227 ± 32) *μ*g/mL. Honghua had an inhibitory effect on *α*-amylase activity within the concentration range (125–1000) *μ*g/mL, but Huangqi did not show any inhibitory effect on *α*-amylase at the tested concentrations (500–1000) *μ*g/mL. The inhibitory effect of Honghuawas significantly lower than acarbose at 500 *μ*g/mL (16.5 ± 5.7% vs. 81.3 ± 7.2%, *P* < 0.01). The inhibitory effect on *α*-amylase at 2 : 1 was significantly higher than that at 20 : 1, 10 : 1, and 5 : 1 at 500 *μ*g/mL and 1000 *μ*g/mL (*P* < 0.01), and 5 : 1 showed a significant higher inhibition effect compared with 20 : 1 and 10 : 1 at 1000 *μ*g/mL (*P* < 0.01).

## 4. Discussion

Acarbose is the first hypoglycemic agent mainly used for controlling postprandial blood glucose and also the first *α*-glucosidase inhibitor approved by the United States Food and Drug Administration (FDA). It is considered to be more suitable for Chinese patients with abnormal glucose metabolism on a carbohydrate-dominated diet and has become one of the most common oral hypoglycemic agents used clinically in China [11]. The main effect of acarbose is to postpone the breakdown of disaccharides and oligosaccharides into glucose, thereby reducing postprandial blood glucose.

China was one of the first countries to recognize diabetes mellitus. The book “*Huangdi Neijing,*” written about 2,000 years ago, has systematically described diabetes mellitus and its complications. A variety of herbs and extracts have been clinically proven to be effective against diabetes mellitus [12]. Some progress has been made in the search for *α*-glucosidase inhibitors from traditional Chinese herbs [13]. This current study aimed to further this investigation.

The treatment of diabetes mellitus with traditional Chinese medicines attaches importance to treatment upon syndrome differentiation so as to provide a guide for clinical medication. According to some research,* qi* deficiency and blood stasis are the main causes and mechanism of diabetes [14]. The traditional Chinese medicines which can supplement* qi*, activate blood circulation, and remove blood stasis are considered to play an important role in the treatment of diabetes mellitus [3].

In traditional Chinese medicine,* qi* and blood, two essential substances for life activities, originate from the viscera and flow constantly inside the body. One role of* qi* is propelling.* Qi* can stimulate and maintain the physiological functions of the viscera and other organs [15]. Deficiency of* qi* in promotion will lead to reduced function and cause various deficiency problems. One class of Chinese medicines is called restoratives for invigorating* qi*, which can tonify the* qi* of the general body to strengthen the functional activity of the body. These restoratives are mainly used for symptoms due to* qi* deficiency. There are many herbs in this class, such as* Radix Ginseng *(Renshen),* Radix Panacis Quinquefolii* (Xiyangshen), and Huangqi [16]. Huangqi is the root of* Astragalus membranaceus *(Fisch.) Bunge var.* mongholicus* (Bunge) Hsiao and* A. membranaceus* (Fisch.) Bunge, family Leguminosae. It mainly grows in Inner Mongolia, Shanxi, Gansu, and Heilongjiang. Its main action is to replenish* qi* [16]. Some research reported that herbs with the action of “invigorating* qi*” are effective in increasing the content of the hepatic cell membrane insulin mediator and improving the sensitivity to serum insulin in diabetic rat models with spleen-*qi* deficiency syndrome [17]. Further research showed that high Huangqi dosages in Buzhong Yiqi decoction can improve the anaerobic oxidative metabolism and regulate the glucose level of rats with* qi* deficiency [18].

As* qi* acts directly to facilitate blood circulation, deficiency of* qi* makes it difficult for* qi* to propel the blood, eventually causing blood stasis [15]. Huangqi with the function of “replenishing* qi*” is always used with medicine with the function for “invigorating the blood and removing blood stasis,” such as Honghua [16]. Huangqi combined with Honghua has been used clinically to treat diabetes mellitus and its complications [4]. Honghua is from the flower of* Carthamus tinctorius* L., family Compositae, and medicinal material is mainly produced in the areas of Henan, Hubei, Sichuan, and Zhejiang [16]. It was reported that some serotonin derivatives found in the seed of* Carthamus tinctorius *L. acted as *α*-glucosidase inhibitors [19]. Our previous research showed that Honghua had an inhibitory effect on yeast *α*-glucosidase activity [20].

Clinical data confirm that acarbose can ameliorate the tendency to blood hypercoagulability in patients, and this study provides evidence that cardiovascular benefits can be obtained from the control of postprandial blood glucose [21]. Huangqiand Honghua are both commonly used clinically to supplement* qi* and activate blood circulation. In this study the effects of mixtures of different proportions of these two on *α*-glucosidase activity were observed using acarbose as a positive control.

Using 4-MUG as a reaction substrate, the product 4-MU obtained after hydrolysis of 4-MUG by yeast *α*-glucosidase was determined by fluorescence analysis. The results showed that acarbose inhibited yeast *α*-glucosidase activity within the concentration range of 0.3–2.5 *μ*g/mL, with an IC_50_ value of 1.8 ± 0.4 *μ*g/mL. Both Huangqiand Honghuaalone inhibited *α*-glucosidase activity; the IC_50_ value of Honghuawas 32.8 ± 5.7 *μ*g/mL, significantly lower than the IC_50_ value of Huangqi of 1686 ± 810 *μ*g/mL.

Studies have found that the glycohydrolase inhibitors from Chinese herbs tend to inhibit both *α*-glucosidase and *α*-amylase [13]. Studies showed that a coffee extract that had an inhibitory effect on *α*-amylase could inhibit the increase in blood glucose due to high starch load in rats, and the extract also had a strong inhibitory effect on *α*-glucosidase [22]. It was reported that acarbose could inhibit pancreatic *α*-amylase, but the inhibitory effect was relatively weak [23]. This study also showed an inhibitory effect of acarbose on porcine pancreatic *α*-amylase, with an IC_50_ value of 227 ± 32 *μ*g/mL, which was significantly higher than that for inhibiting *α*-glucosidase, with a 126-fold difference between them. A similar result was obtained for Honghua: at the same active concentration of 125 *μ*g/mL it showed 89.8 ± 5.6% inhibition of *α*-glucosidase, but only 5.8 ± 2.1% inhibition of *α*-amylase (Tables 2 and 3). Huangqidid not show any effect on *α*-amylase in the tested concentration range of 500–1000 *μ*g/mL. In terms of simultaneously inhibiting *α*-glucosidase and *α*-amylase activity, Honghua,which activates blood circulation and removes blood stasis, seemed significantly better than Huangqi,which supplements* qi*.

Studies have shown that the main components in traditional Chinese medicines for treating diabetes mellitus belong to four categories: saponins, flavonoids, polysaccharides, and alkaloids [24]. The saponin astragaloside IV in Huangqiinjection and total flavonoids in Honghua injection were quantitatively monitored as the main active ingredients in the respective preparations. According to the national standards established by CFDA, Huangqiinjection should contain no less than 0.08 mg of astragaloside IV per mL [7], and the content of astragaloside IV in this study was 0.127 mg/mL; Honghuainjection should contain 0.20–0.70 mg of total flavonoids on the basis of kaempferol equivalents per mL [8], and the content in this study was 0.57 mg/mL. In this study, their proportions for raw herbs and injections in clinical applications were based on the ratios of their active ingredients (Table 1).

In clinical applications, proportions of Huangqiand Honghuainjection ranging from 4 : 1 to 10 : 1 on the basis of raw herbs have been reported [4–6]. In this study, we designed a wider range of preparations, that is, four groups of different proportions, 20 : 1, 10 : 1, 5 : 1, and 2 : 1. The results showed that the yield of the Honghuainjectionextract was 10.44%, which was 10 times greater than the extraction yield of Huangqi, 1.04%, and the extraction yield of total flavonoids in Honghua injection was 20 times greater than that of astragaloside IV in Huangqi injection. This probably was one of the reasons why the clinical usage amount of Huangqi injection was greater than that of Honghua injection.

**Figure 1:**
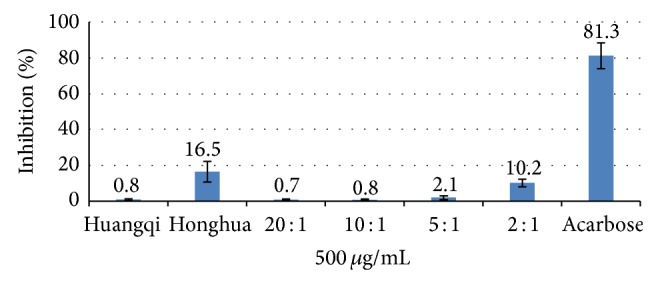
Inhibition of acarbose, Huangqi (*Radix Astragali Mongolici*) injection, Honghua (*Flos Carthami*) injection, and different proportions on *α*-amylase activity at 500 *μ*g/mL. *P* < 0.01, proportions 20 : 1, 10 : 1, and 5 : 1 compared with 2 : 1 at 500 *μ*g/mL.

**Figure 2:**
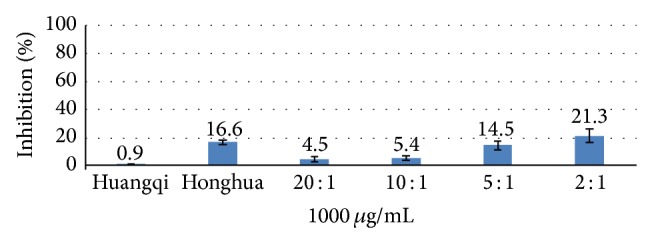
Inhibition of Huangqi (*Radix Astragali Mongolici*) injection, Honghua (*Flos Carthami*) injection, and different proportions on *α*-amylase activity at 1000 *μ*g/mL. *P* < 0.01, proportions 20 : 1, 10 : 1, and 5 : 1 compared with 2 : 1 at 1000 *μ*g/mL and proportions 20 : 1, 10 : 1 compared with 5 : 1 at 1000 *μ*g/mL.

IC_50_s on *α*-glucosidase at 10 : 1, 5 : 1, and 2 : 1 were significantly lower than IC_50_ at 20 : 1 (*P* < 0.01). The inhibitory effect on *α*-amylase at 2 : 1 was significantly higher than that at 20 : 1, 10 : 1, and 5 : 1 at 500 *μ*g/mL, 1000 *μ*g/mL separately (*P* < 0.01) (Figures 1 and 2). Proportion 5 : 1 showed a significantly higher inhibitory effect compared with 20 : 1 and 10 : 1 at 1000 *μ*g/mL (*P* < 0.01) (Figure 2). In terms of simultaneously improving the above two indicators, the raw herbs proportions of 2 : 1 and 5 : 1 exhibited significant effects compared to those of 20 : 1.

In this study, the four groups of different proportions were superior to Huangqialonebut inferior to Honghua alonefor the inhibition of *α*-glucosidase activity. The combination of Huangqi and Honghua injections was reported to be more effective than Honghua injection alone for inhibiting apoptosis of nerve cells around a cerebral hemorrhage in rats [25]. There did not appear to be a synergistic effect in this research.* In vivo* experiments may be required to better understand and optimize the herbal formulation.

## Figures and Tables

**Table 1:** Different mixture proportions of Huangqi (*Radix Astragali Mongolici*) and Honghua (*Flos Carthami*).

Proportions of raw herb	Huangqi injection^*^ (mL)	Honghua injection^#^ (mL)	Proportions of extract	Proportions of astragaloside IV and total flavonoids
20 : 1	10	2	2 : 1	1.1 : 1
10 : 1	10	4	1 : 1	0.5 : 1
5 : 1	10	8	0.5 : 1	0.25 : 1
2 : 1	10	20	0.2 : 1	0.1 : 1

Notes: ^*^Huangqi injection contained 0.5 g of raw drug per mL. ^#^Honghua injection contained 2 g of raw drug per mL.

**Table 2:** Inhibitory effect of Huangqi (*Radix Astragali Mongolici*) injection, Honghua (*Flos Carthami*) injection, and different proportions on yeast *α*-glucosidase activity ($\overset{-}{x}\;\pm \;s$).

Group	Concentration (*µ*g/mL)	Percentage of inhibition (%)	IC_50_ (*µ*g/mL)
Acarbose	2.5	70.2 ± 3.2	1.8 ± 0.4
	1.3	34.0 ± 2.7	
	0.6	24.4 ± 1.7	
	0.3	12.9 ± 1.2	

Huangqi injection	2000	54.1 ± 5.6	1686 ± 810^a^
	1500	43.6 ± 3.7	
	750	38.1 ± 2.6	
	375	9.7 ± 1.1	

Honghua injection	125	89.8 ± 5.6	32.8 ± 5.7^ab^
	62.5	66.9 ± 5.1	
	31.3	46.6 ± 3.7	
	15.6	22.8 ± 3.1	

Different proportions			
20 : 1	250	67.5 ± 5.7	122.6 ± 21.4^ab^
	125	49.9 ± 5.1	
	62.5	29.1 ± 3.1	
	31.3	27.3 ± 2.9	
10 : 1	250	82.0 ± 7.6	53.8 ± 5.5^abc^
	125	67.8 ± 5.3	
	62.5	57.3 ± 5.5	
	31.3	34.8 ± 3.3	
5 : 1	250	88.1 ± 9.1	55.5 ± 7.2^abc^
	125	77.5 ± 6.9	
	62.5	48.8 ± 4.1	
	31.3	35.3 ± 2.1	
2 : 1	250	85.7 ± 8.1	52.4 ± 11.6^abc^
	125	66.8 ± 6.1	
	62.5	56.9 ± 6.1	
	31.3	33.2 ± 3.0	

Notes: ^a^ *P* < 0.001, compared with acarbose control. ^b^ *P* < 0.001, Honghua injection and different proportions compared with Huangqi injection. ^c^ *P* < 0.01, proportions 10 : 1, 5 : 1, and 2 : 1 compared with 20 : 1. IC_50_: half maximal (50%) inhibitory concentration on yeast *α*-glucosidase activity.

**Table 3:** Inhibitory effect of Huangqi (*Radix Astragali Mongolici*) injection, Honghua (*Flos Carthami*) injection, and different proportions on *α*-amylase activity ($\overset{-}{x}\pm s$).

Group	Concentration (*µ*g/mL)	Percentage of inhibition (%)
Acarbose	500	81.3 ± 7.2
	250	65.7 ± 7.1
	125	32.1 ± 4.7
	62.5	24.4 ± 1.9

Huangqi injection	1000	0.9 ± 0.4
	500	0.8 ± 0.5

Honghua injection	1000	16.6 ± 1.4^c^
	500	16.5 ± 5.7^ab^
	250	6.9 ± 1.3
	125	5.8 ± 2.1

Different proportions		
20 : 1	1000	4.5 ± 1.7
	500	0.7 ± 0.5
10 : 1	1000	5.4 ± 1.8
	500	0.8 ± 0.5
5 : 1	1000	14.5 ± 3.2
	500	2.1 ± 1.0
2 : 1	1000	21.3 ± 4.6
	500	10.2 ± 2.1

Notes: ^a^ *P* < 0.01, Honghua injection compared with acarbose at 500 *µ*g/mL. ^b^ *P* < 0.01, Honghua injection compared with Huangqi injection at 500 *µ*g/mL. ^c^ *P* < 0.01, Honghua injection compared with Huangqi injection at 1000 *µ*g/mL.
